# Supplementary material for: Evaluation of anti-ulcer activity of hydromethanol crude extract and solvent fractions of Vicia faba (Fabaceae) seeds in mice
Source: PLoS One. 2025 Oct 9;20(10):e0334119. doi: 10.1371/journal.pone.0334119 (PMC12510542; doi:10.1371/journal.pone.0334119)
Supplement: Attachment — Submitted filename: Plose one manuscript comment.docx [file pone.0334119.s002.docx]

- **I found that the topic was interesting because I am one of individuals suffered with gastric ulcers!**
- **Comments for authors**
- Vicia faba should be italicized throughout the manuscript
- Line 115: What plant material was identified by the botanist? B/c you have bought the *Faba bean* seeds from market…..line113?
- muslin cloth??........line137
- Line 168: and there were no dead mice or observed signs of toxicity; parts of result
- Line 173: plant extract/seed extract???
- Line 199; After four h. of pyloric ligation?
- Laboratory study period should be included?
- Line 411-19: discussion portion irrelevant?? Discuss you finding with previous work rather than introducing methods and objectives?
- Line 507-518: Conclusion: *V. faba* seeds crude extract and solvent fractions have significant antiulcer activity administered at single and repeated doses …..at what concentrations??
- **Important question:**
- Mostly the Ethiopian community utilizes *Faba bean* for consumption in cooked form rather than raw seed but your experiment involves raw powdered *Faba bean* against laboratory mice? Can heat affect the peptic ulcer healing property of *Faba bean*/not?? B/c you observed the uncooked powdered *Faba bean* seeds?
