# Supplementary material for: Evaluation of anti-ulcer activity of hydromethanol crude extract and solvent fractions of Vicia faba (Fabaceae) seeds in mice
Source: PLoS One. 2025 Oct 9;20(10):e0334119. doi: 10.1371/journal.pone.0334119 (PMC12510542; doi:10.1371/journal.pone.0334119)
Supplement: Attachment — Submitted filename: Responses to Reviewers.docx [file pone.0334119.s004.docx]

**Subject:** Submission of Point-by-Point Response for Manuscript ID **PONE-D-24-53911**

**Dear PLOS ONE Editors**:

I hope this message finds you well. I am writing to submit the **point-by-point response** to the reviewers' comments for **Manuscript ID PONE-D-24-53911**, entitled “Evaluation of Anti-Ulcer Activity of Hydromethanol Crude Extract and Solvent Fractions of Vicia faba (Fabaceae) Seeds in Mice.” Attached to this email, you will find our comprehensive response addressing each of the reviewers' comments. We have carefully considered their feedback and made the necessary revisions to improve the quality and clarity of the manuscript. We appreciate the time and effort the reviewers have dedicated to evaluating our work and providing valuable insights. We believe that the revisions we have made address their concerns effectively. Please do not hesitate to reach out if you require any further information or clarification regarding our response. We look forward to your feedback on the revised manuscript. Thank you for your continued support and guidance throughout the review process.

Sincerely,
**Demis Getachew Shawule**
University of Gondar,
Gondar, Ethiopia
demisgetachew1@gmail.com

Point-by-point correction and response to the reviewer

**General Comments:**

Dear Editors and reviewer

Thank you for the constructive comments. We have revised the manuscript according to the journal's guidelines and comments.

Comments to the Author

**Comments:** 1. Is the manuscript technically sound, and do the data support the conclusions?

The manuscript must describe a technically sound piece of scientific research with data that supports the conclusions. Experiments must have been conducted rigorously, with appropriate controls, replication, and sample sizes. The conclusions must be drawn appropriately based on the data presented.

Reviewer #1: Yes

**Responses:** Thank you

Reviewer #2: Yes

**Responses:** Thank you

**Comments:** 2. Has the statistical analysis been performed appropriately and rigorously?

Reviewer #1: No

**Responses:** We believe the statistical analysis was performed rigorously. We conducted an ANOVA (Analysis of Variance) to assess whether there were significant differences between the groups. Following the ANOVA, We performed a Tukey post-hoc test to determine which specific group pairs differ significantly from one another. Both tests are appropriate for this type of analysis, as ANOVA helps identify overall group differences, while Tukey's test ensures that any pairwise differences are evaluated while controlling for the risk of Type I errors.

Reviewer #2: Yes

**Responses:** Thankyou

**Comments:** 3. Have the authors made all data underlying the findings in their manuscript fully available?

The PLOS Data policy requires authors to make all data underlying the findings described in their manuscript fully available without restriction, with rare exception (please refer to the Data Availability Statement in the manuscript PDF file). The data should be provided as part of the manuscript or its supporting information, or deposited to a public repository. For example, in addition to summary statistics, the data points behind means, medians and variance measures should be available. If there are restrictions on publicly sharing data—e.g. participant privacy or use of data from a third party—those must be specified.

Reviewer #1: No

**Responses:** We think we made sufficient data underlying the findings available in the manuscript. Moreover, the full data set was submitted to the journal as **Supplementary file1** during manuscript submission.

Reviewer #2: Yes

**Responses:** Thank you

**Comments**: 4. Is the manuscript presented in an intelligible fashion and written in standard English?

PLOS ONE does not copyedit accepted manuscripts, so the language in submitted articles must be clear, correct, and unambiguous. Any typographical or grammatical errors should be corrected at revision, so please note any specific errors here.

Reviewer #1: No

**Responses:** Thank you! We have reviewed the entire document and made several changes to fix grammatical errors. We corrected some grammatical errors using QuillBot's online grammar checker. Additionally, Dr. Yoseph, a senior English language professional (PhD in Teaching English as a Foreign Language (TEFL)) at the University of Gondar, revised the document for language clarity, and further corrections were made based on his recommendation.

Reviewer #2: Yes

**Responses:** Thank you

5. Review Comments to the Author

**Comments:** Reviewer #1: While the study suggests potential for developing new herbal medicines with anti-ulcer effects its ethno pharmacological relevance, however, the results are preliminary and the mechanisms of action were not well explored. I do not support it in this instance. However, I suggest further revisions in the manuscript

**Responses:** Thank you for your valuable comments and suggestions. We revised the manuscript accordingly.

**Comments:** î the manuscript has many grammatical and language flaws to be edited with a native speaker.

î I feel concerned with the writing and the English, in which sometimes seems to make no sense.

**Responses:** Thank you for your constructive comments. While we are unable to have a native speaker review the manuscript at this time, as much as possible, we have corrected the grammatical flaws using QuillBot's online grammar checker to improve clarity, coherence, and overall readability. The document was also Dr. Yoseph, a senior English language professional (PhD in Teaching English as a Foreign Language (TEFL)) at the University of Gondar, revised the document for language clarity, and further corrections were made based on his recommendation.

**Comments:** î In preparation of plant seed extract why do you prefer hydromethanol? How is it prepared in the traditional medicine?

**Responses:** We preferred hydromethanol due to its expanded polarity index. Hydroalcoholic solvent combinations are often thought to provide good extraction yields. In general, hydroalcoholic co-solvents like 80% methanol appear to have the best solubility properties for initial crude extraction (Reference; Title: *Antidiarrheal activities of methanolic crude extract and solvent fractions of the root of Verbascum sinaiticum Benth. (Scrophularaceae) in mice; Authors: Solomon Ayenew Worku, Solomon Asmamaw Tadesse, Mohammedbrhan Abdelwuhab, Assefa Belay Asrie*). Furthermore, we found that methanol was used to extract active metabolites of V.faba like polyphenols, and flavonoids (Study conducted in Egypt; Title: *Antioxidant, anti-inflammatory, antimicrobial, and anticancer properties of green broad bean pods (Vicia faba L*.)). In traditional medicine, hydromethanol was not commonly used to prepare Vicia faba, as its users typically chewed the dry seeds directly to treat stomach ulcers. This explanation is removed in the revised manuscript in response to another reviewer’s comment.

**Comments:** î Page 12: Table 1: Qualitative phytochemical analysis of V. faba seeds crude extract and solvent fractions. (I suggest discussing the chemical composition of V. faba after identifying by HPLC?)

**Responses:** Thank you for your feedback. We wholeheartedly acknowledge the necessity and significance of performing HPLC analysis to identify bioactive chemicals with antiulcer activity. However, due to our institution's lack of laboratory facilities that would have allowed us to conduct the research, we could not include data in this respect. We formulated the study's goals from the start, taking into account the available resources and laboratory facilities that can make our plans feasible. Otherwise, we have acknowledged this point in full and do not question the significance of conducting a more sophisticated study that might provide more precise information about the bioactive chemicals that may have an antiulcer effect. As a result, we listed this concern as one of the study's limitations and suggested that more research be done on the plant's chemical constituent and potential molecular mechanisms of action to explain its observed antiulcer effect.

**Comments:** î What do you attribute this reduction to? Chemistry composition?

**Responses:** Thank you for your feedback. The reduction to this is attributed to the lack of facilities including HPLC and the chemicals that are used for HPLC function.

**Comments:** î Stress a notorious cause of ulcer was not represented in this study. Do you come up with a clear reason?

Responses: Thank you for your valuable comment. The omission of stress as a cause of ulcers in this study was intentional. Since this manuscript is part of a thesis, we aimed to keep the focus narrow and concise. As such, we intentionally excluded stress as the third most important stress-inducing factor. However, we have now addressed its role in the pathogenesis of ulcers in the revised manuscript. Please refer to the revised manuscript.

**Comments:** î The authors should describe how doses were chosen for the study

**Responses:** Thank you for your deep insight and comment**.**  The test doses were determined by taking 1/20th of the limit test of the extract as the low dose, 1/10th of the limit test of the extract as the middle dose, and 1/5th of the limit test of the extract as the third dose, according to OECD 425 guideline. We explained this in the revised manuscript.

î Due to English language issues results are hard to follow.

î On the whole, practically no discussion was elaborated as mere results were just mentioned again and again. In many part of the discussion, the authors discussed many phytochemicals which they never studied in this work. The preliminary phytochemical study cannot be used as a justification, since, as the name suggests and which it is is 'preliminary', there is need to do fingerprinting of the plant or at least quantification of some of the important phytoconstituents like polyphenols, flavonoids etc.

Thank you

Reviewer #2:

I found that the topic was interesting because I am one of individuals suffered with gastric ulcers!

Comments for authors

**Comments:** Vicia faba should be italicized throughout the manuscript

**Responses:** Thank you for your constructive feedback and insightful comments. We have italicized “*Vicia faba*” throughout the revised manuscript accordingly.

**Comments:** Line 115: What plant material was identified by the botanist? B/c you have bought the Faba bean seeds from market…..line113?

**Responses**: Thank you for your constructive comments. First, we brought the seed to a Botanist for identification. Then, the botanist ordered us to cultivate the plant as the whole plant was not available when we were collecting the experimental plant. Accordingly, we brought the seed and the whole plant after cultivating it, and the plant material (the whole plant) was identified and authenticated by Mr. Getenet Chekole, (Assistant Professor of Botanical Science in the Department of Biology at the College of Natural and Computational Sciences, University of Gondar). Subsequently, specimens were deposited at the University of Gondar herbarium with voucher 215/07/2024.

**Comments:** muslin cloth??........line137

**Responses:** Thank you for your feedback. Muslin cloth is a loosely woven, cotton fabric that is effective in separating solid particles from liquids. When filtering crude extracts (for example, in the preparation of plant extracts, oils, or juices), muslin cloth can be used to strain out larger solid materials, leaving behind the finer, liquid components. So, we use it in this experiment to separate large solid particles of the macerated bean seed powder from the liquids followed by filter paper filtration.

**Comments:** Line 168: and there were no dead mice or observed signs of toxicity; parts of result

**Responses:** Thank you for your deep insight and comment. We have put it in the result section. See the result section of the revised manuscript.

**Comments:** Line 173: plant extract/seed extract???

**Responses:** Thank you for your deep insight and comment. It is a writing mistake, we corrected it as “seed extract”.

**Comments:** Line 199; After four h. of pyloric ligation?

**Responses:** Thank you for your deep insight and comment. It is a writing mistake. We corrected it as hours

**Comments:** Laboratory study period should be included?

**Responses:** Thank you for your feedback. We have included the study period accordingly.

**Comments:** Line 411-19: discussion portion irrelevant?? Discuss you finding with previous work rather than introducing methods and objectives?

**Responses:** Thank you for your feedback. We corrected it accordingly. See the revised manuscript.

**Comments:** Line 507-518: Conclusion: V. faba seeds crude extract and solvent fractions have significant antiulcer activity administered at single and repeated doses …..at what concentrations??

**Responses:** Thank you for your comments and suggestions. Significant antiulcer activity administered at single and repeated doses of *V. faba* seeds was observed at 200 and 400 mg/kgs.

**Comments:** Important question:

Mostly the Ethiopian community utilizes Faba bean for consumption in cooked form rather than raw seed but your experiment involves raw powdered Faba bean against laboratory mice? Can heat affect the peptic ulcer healing property of Faba bean/not?? B/c you observed the uncooked powdered Faba bean seeds?

 Important points to be considered:

 Mostly the Ethiopian community utilizes Faba bean for consumption in cooked form rather than raw seed but your experiment involves raw powdered Faba bean against laboratory mice? Can heat affect the peptic ulcer healing property of Faba bean/not?? B/c you observed the uncooked powdered Faba bean seeds?

**Responses:** Thank you for your important comments. Mostly the Ethiopian community utilizes Faba bean for consumption in cooked form rather than raw seed. However, for treatment purposes, they directly chew the dried seed. For example, you can see the ethnobotanical studies that we cited:

**Title:** An ethnobotanical study of medicinal plants in Wayu Tuka District, East Welega Zone of Oromia Regional State, West Ethiopia.

**Authors:** Moa Megersa, Zemede Asfaw, Ensermu Kelbessa, Abebe Beyene, and Bizuneh Woldeab

**Title:** Ethnobotanical study of medicinal plant species in Nensebo District, south-eastern Ethiopia

**Authors**: Gemedi Abdela, Zerihun Girma and Tesfaye Awas
