# Supplementary material for: Evaluation of anti-ulcer activity of hydromethanol crude extract and solvent fractions of Vicia faba (Fabaceae) seeds in mice
Source: PLoS One. 2025 Oct 9;20(10):e0334119. doi: 10.1371/journal.pone.0334119 (PMC12510542; doi:10.1371/journal.pone.0334119)
Supplement: Attachment — Submitted filename: Responses_to_Reviewers_auresp_2.docx [file pone.0334119.s005.docx]

**Subject:** Submission of Point-by-Point Response for Manuscript ID **PONE-D-24-53911R1**

**Dear PLOS ONE Editors**:

I hope this message finds you well. I am writing to submit the **point-by-point response** to the reviewers' comments for **Manuscript ID PONE-D-24-53911R1**, entitled “Evaluation of Anti-Ulcer Activity of Hydromethanol Crude Extract and Solvent Fractions of Vicia faba (Fabaceae) Seeds in Mice.” Attached to this email, you will find our comprehensive response addressing each of the reviewers' comments. We have carefully considered their feedback and made the necessary revisions to improve the quality and clarity of the manuscript. We appreciate the time and effort the reviewers have dedicated to evaluating our work and providing valuable insights. We believe that the revisions we have made address their concerns effectively. Please do not hesitate to reach out if you require any further information or clarification regarding our response. We look forward to your feedback on the revised manuscript. Thank you for your continued support and guidance throughout the review process.

Sincerely,
**Demis Getachew Shawule**
University of Gondar,
Gondar, Ethiopia
[demisgetachew1@gmail.com](mailto:demisgetachew1@gmail.com)

Point-by-point correction and response to the reviewer

**General Comments:**

Dear Editors and reviewer

Thank you for the constructive comments. We have revised the manuscript according to the journal's guidelines and comments.

Comments to the Author

**Additional Editor Comments:**

Dear Authors,

Although some reviewers have recommended acceptance of the manuscript, please note that this does not constitute a final editorial decision. The editorial team has carefully reviewed the reviewers’ comments and your responses.
**Comments:** Before proceeding further, we kindly request that you thoroughly address all reviewers’ comments, with special attention to the concerns raised by Reviewer 1 and Reviewer 3.

**Responses:** Thank you for your suggestions. We have addressed all the reviewer comments accordingly.

**Comments:** Reviewer 1 highlighted important issues regarding the preparation of the product and the phytochemical analysis, which were not sufficiently addressed. Reviewer 3 also provided several significant comments that still require revision, despite their general recommendation to accept the manuscript.
**Responses:** Thank you for your suggestions. We have addressed Reviewer 3's comments accordingly. You can see in the revised manuscript. However, we didn’t find the reviewer 1's comments, and you have also informed us that he is unavailable.
**Comments:** Please revise the manuscript accordingly and ensure that each comment is clearly and individually responded to in a point-by-point response letter.

**Responses:** Thank you. We have addressed all the comments accordingly

**Review Comments to the Author**

**Comments**: **Reviewer #2:** Comments incorporated! You have selected appropriate area for investigation. Try to to bring the finding of this research to the community.

**Responses:** Thank you for your encouraging feedback. We appreciate your positive evaluation of the research area we selected. In response to your suggestion, we have outlined possible strategies for disseminating the findings through academic presentations, open-access publications, and community-focused workshops.

**Comments: Reviewer #3**: This manuscript presents a meaningful study on the anti-ulcer potential of Vicia faba seed extracts using both ethanol-induced and pylorus ligation models in mice. The experimental design is sound, and the findings support the plant’s traditional use in treating gastric ulcers. The inclusion of both single and repeated dosing regimens is commendable and strengthens the validity of the results.
That said, a few minor points should be considered:
**Comments:** 1.While the study shows promising results, a brief discussion of potential mechanisms (e.g., antioxidant or mucosal protection) would enhance the paper.

**Responses:** Thank you for your Suggestions. We have briefly discussed the mechanism of action accordingly. See the revised manuscript.

**Comments:** 2.The ulcer severity assessment is based only on visible observation. Adding tissue-level analysis would help support the findings more convincingly. The lack of histological analysis is understandable but should be acknowledged as a limitation.

**Responses:** Thank you for your valuable comments. We fully agree that the addition of histological analysis would have strengthened our findings. Due to resource constraints, we were unable to perform tissue-level histological evaluations in the current study. However, we have now explicitly acknowledged this as a limitation in the Strengths and Limitations section of the revised manuscript.

**Comments:** 3.Some language polishing is still needed to improve clarity and flow.

**Responses:** We thank the reviewer for pointing out the need for improved language clarity and flow. In response, we have thoroughly revised the manuscript to enhance its readability and coherence. Grammatical errors and awkward phrasings have been corrected, and sentence structures have been refined for better clarity. We also improved transitions between sections and ensured consistency in terminology throughout the text. We believe these changes have significantly improved the overall presentation and quality of the manuscript.
**Comments:** 4.A short comment on how the extraction method compares to traditional use would provide helpful context.

In traditional medicine, hydromethanol was not commonly used to prepare *Vicia faba*, as its users typically chewed the dry seeds directly to treat stomach ulcers (stated in the introduction section of the manuscript). But, we selected based on previous studies that methanol was used to extract active metabolites of *V.faba,* like polyphenols, and flavonoids (Study conducted in Egypt; Title: Antioxidant, anti-inflammatory, antimicrobial, and anticancer properties of green broad bean pods (Vicia faba L.)). This explanation is removed in the revised manuscript in response to another reviewer’s comment.

**Responses:**
Accept with minor edits. The study is valuable and well-conducted. With slight improvements in presentation and clarity, it will make a good contribution to the field.

**Comments: Reviewer #4:** The manuscript has been revised by authors in accordance with reviewers’ comments and suggestions. As per suggestion, the revised manuscript has been supplemented with further relevant information. Various sections particularly experimental as well as discussion are modified for better clarity. The grammatical and linguistic errors have been rectified and presentation of text matter has been improved. Based on the work described, scope of further research is mentioned. Other suggested corrections/modifications have also been aptly incorporated and a detailed clarification has been submitted by author(s) against various queries of reviewers.

**Responses**: Thank you for the thorough evaluation and constructive feedback.

**Comments: Reviewer #5:** This is an interesting study conducted by the authors in a scientifically sound manner. The study design is clearly outlined as well as the details of the mice models that were used and the methods used for inducing ulcers. The protocols are clearly outlined and in great detail. The use of tables further adds to the visual quality of the manuscript. However some of the graphs were mentioned in the supplementary data and would be helpful if they are included in the main text itself as that will further and hence the quality of the manuscript. The statistical methods are described in very brief detail, further elaboration would also be helpful. The authors have used appropriate and relevant references to support their study and their discussion. They have also outlined the strength in limitations of the study separately as well.

**Responses:** Thank you for the encouraging and thoughtful feedback on our manuscript.
