# Supplementary material for: Evaluation of anti-ulcer activity of hydromethanol crude extract and solvent fractions of Vicia faba (Fabaceae) seeds in mice
Source: PLoS One. 2025 Oct 9;20(10):e0334119. doi: 10.1371/journal.pone.0334119 (PMC12510542; doi:10.1371/journal.pone.0334119)
Supplement: Attachment — Submitted filename: Responses_to_Reviewers_auresp_3.docx [file pone.0334119.s006.docx]

**Subject:** Submission of Point-by-Point Response for Manuscript ID **PONE-D-24-53911R2**

**Dear PLOS ONE Editors**:

I hope this message finds you well. I am writing to submit the **point-by-point response** to the reviewers' comments for **Manuscript ID PONE-D-24-53911R2**, entitled “Evaluation of Anti-Ulcer Activity of Hydromethanol Crude Extract and Solvent Fractions of Vicia faba (Fabaceae) Seeds in Mice.” Attached to this email, you will find our comprehensive response addressing each of the reviewers' comments. We have carefully considered their feedback and made the necessary revisions to improve the quality and clarity of the manuscript. We appreciate the time and effort the reviewers have dedicated to evaluating our work and providing valuable insights. We believe that the revisions we have made address their concerns effectively. Please do not hesitate to reach out if you require any further information or clarification regarding our response. We look forward to your feedback on the revised manuscript. Thank you for your continued support and guidance throughout the review process.

Sincerely,
**Demis Getachew Shawule**
University of Gondar,
Gondar, Ethiopia
demisgetachew1@gmail.com

Point-by-point correction and response to the reviewer

**General Comments:**

Dear Editors and reviewers

Thank you for the constructive comments. We have revised the manuscript according to the journal's guidelines and comments.

Additional Editor Comments:

**Comments:** 1. Dear Authors,

Thank you for your revised submission to PLOS ONE. We acknowledge the improvements you have made in response to the reviewers’ comments. However, after further consideration, we find that additional refinement is still required before the manuscript can move forward.

**Comments:** In particular, while you have revised the Discussion and Conclusion sections, the current phrasing still requires careful adjustment. The conclusions should be presented in a way that accurately reflects the scope of the study, which is limited to animal experiments. Generalizations regarding the efficacy of Vicia faba seeds in humans should be avoided, and the text should instead clearly emphasize the study’s limitations and the need for well-designed clinical investigations to confirm these findings.

Comments: We therefore ask that you:

Further refine the Discussion to provide a balanced interpretation of the results without overstatement.

**Responses:** Thank you for your valuable feedback. We have carefully revised the Discussion and Conclusion sections to ensure that the statements accurately reflect the scope of our study. We have avoided generalizations regarding the efficacy of *Vicia faba* seeds in humans and instead emphasized the study’s limitations. The revised text clearly highlights that the findings are restricted to animal experiments and underscores the need for well-designed clinical studies to validate these results.

**Comments:** Adjust the Conclusion to ensure consistency with the presented data and to highlight the preliminary nature of the findings.

These refinements will ensure that your manuscript aligns with the journal’s publication standards and will significantly improve its clarity and scientific value. We appreciate your efforts and look forward to receiving a carefully revised version.

**Responses:** We thank the reviewer for this important suggestion. The Conclusion section has been revised to ensure consistency with the presented data and to highlight the preliminary nature of the findings. The revised text now clearly states that the results are based on animal experiments and should be interpreted as preliminary, with further well-designed clinical studies required to validate these observations
